# Approbation of a New Model of Secondary Damage after Traumatic Brain Injury Based on Reprogrammed Rat Embryo Fibroblasts

**DOI:** 10.1134/S1607672923700345

**Published:** 2023-10-13

**Authors:** E. B. Rykunova, M. A. Mikeladze, I. A. Utepova, O. N. Chupakhin, I. V. Guzhova, V. F. Lazarev

**Affiliations:** 1 Institute of Cytology, Russian Academy of Sciences, St. Petersburg, Russia; 2 Ural Federal University, Yekaterinburg, Russia; 3 Institute of Organic Synthesis, Ural Branch, Russian Academy of Sciences, Yekaterinburg, Russia

**Keywords:** traumatic brain injury, cerebrospinal fluid, secondary injuries, chaperones, Hsp70, pyrrolylazine and indolylazine derivatives

## Abstract

The paper presents a new model of secondary injuries after traumatic brain injury. The model is based on the cultivation of rat embryonic fibroblasts reprogrammed to a neuronal phenotype in the presence of cerebrospinal fluid from injured rats. The presented model was used to test the therapeutic effect of inducers of the synthesis of chaperones from the classes of pyrrolylazines and indolylazines, which have neuroprotective properties.

Traumatic brain injury (TBI) may cause long-term neuronal death delayed for months. This phenomenon is called secondary damage and is traditionally associated with changes in blood supply, ischemia, hypoxia, inflammation, cerebral edema, increased intracranial pressure, etc. [12]. At the molecular level, secondary damage after TBI can be caused by the generation of reactive oxygen species, excitotoxicity, and mitochondrial dysfunction, which ultimately leads to the death of glial and neuronal cells, most often through the mechanisms of apoptosis [3] or necrosis [4]. Potentially toxic cell-death products can accumulate in the cerebrospinal fluid (CSF), because they are transported from the interstitial fluid through the glymphatic system [5].

Many scientific groups are involved in modeling secondary injuries after TBI in vitro. This is determined by the need to be able to test new therapeutic approaches or potential therapeutic agents without using animal models. Almost always modeling is reduced to simulating the effect of a single factor that causes secondary damage, for example, oxidative stress [6] or inflammatory response [7], which does not reflect the entire spectrum of pathological processes.

In our previous studies, we developed and tested a new model for analyzing secondary damage after traumatic brain injury based on cells cultured in the presence of CSF of injured animals [8]. The advantages of our model were that it simultaneously took into account many factors that determined secondary damage and made it possible to test potential therapeutics by adding them to the growth medium of cells containing the cerebrospinal fluid of injured animals. The main drawback of the previously used models was that we used tumor cells (C6 rat glioblastoma) as acceptor cells. In view of this, the main objective of this study was to develop a relevant model of secondary damage after brain injury based on the culture of rat cells with a neuronal phenotype.

Rat embryonic fibroblasts DFK-3 were obtained from the Vertebrate Cell Culture Collection core facility, which was supported by a grant from the Ministry of Education and Science of the Russian Federation (agreement no. 075-15-2021-683). Cells were cultured in DMEM medium (BioloT, Russia) supplemented with 10% fetal bovine serum (Gibco, United States) and antibiotics penicillin 100 units/mL and streptomycin 0.1 mg/mL (BioloT, Russia) at 37°C and 5% CO_2_.

These cells were reprogrammed into a neuronal phenotype (DFK3-Neu) by incubating them for 5 days in the Neurobasal medium (BioinnLabs, Russia) containing the Neuromax supplement (PanEco, Russia), 3% fetal bovine serum, 100 units/mL penicillin, and 0.1 mg/mL streptomycin. The neuronal phenotype was tested by analyzing the expression of a panel of markers of mature neurons [9, 10] (NeuN, MAP2, 160 kDa neurofilament medium (NF-M), 200 kDa neurofilament heavy (NF-H), Synaptophysin (Syn), and PSD95 using real-time PCR. For this purpose, cell lysates collected from an area of 27 cm^2^ were dissolved in 600 µL of the ExtractRNA reagent (Evrogen, Russia). mRNA was isolated according to the previously described protocol [11]. Then, to obtain cDNA, a reverse transcription reaction was performed using the MMLV RT kit (Evrogen, Russia) according to the manufacturer’s protocol. For analysis, 2 μg of RNA per sample was used.

All real-time PCR reactions were performed in a CFX96 real-time PCR detection system (BioRad, United States) using qPCRmix-HS SYBR (Evrogen, Russia) according to the manufacturer’s protocol. Data were analyzed for fold change in mRNA using Bio-Rad CFX software (version 3.1). Nucleotide sequences of primers are given in Table 1. GAPDH was used as a normalization control. All primers were ordered from Evrogen (Russia). Real-time PCR was performed under the following conditions: 5 min pre-denaturation at 95°C and then 30 s at 95°C, 30 s at 62°C, and 30 s at 72°C (40 cycles).

**Table 1.** Primer sequences used in the study

GAPDH	Forward	5'-ATGATTCTACCCACGGCAAG-3'
	Reverse	5'-CTGGAAGATGGTGATGGGTT-3'
NeuN	Forward	5'-GGCTGCTGATCCCTACCATC-3'
	Reverse	5'-GAAGCGGCTGTACCCTCC-3'
MAP2	Forward	5'-CAACACAAGGATCAGCCCTGC-3'
	Reverse	5'-TGTTTGTTCTGATGCTGGCG-3'
NF-M	Forward	5'-TCCTCAGTCCTTGGGGGAAT-3'
	Reverse	5'-TGCCCCTCTTTCAACAGCTT-3'
NF-H	Forward	5'-CCAGGATGCAATTCAGCAGC-3'
	Reverse	5'-TCTTGACGTTGAGCAGGTCC-3'
Syn	Forward	5'-TCGTGTTCAAGGAGACAGGC-3'
	Reverse	5'-CAGGTGCTGGTTGCTTTTCC-3'
PSD95	Forward	5'-AAGATGAAGACACGCCCCC-3'
	Reverse	5'-ATCACAGGGGGAGAATTGGC-3'

We found that the expression level of the genes encoding the main neuronal markers in DFK3-Neu cells (embryonic fibroblasts after neuronal differentiation) was, on average, 2–4 times higher than their expression level in DFK3 cells (Fig. 1). An increase in the expression of mature neuron markers indicates the acquisition of a neuronal phenotype by cells [9].

**Fig. 1.**
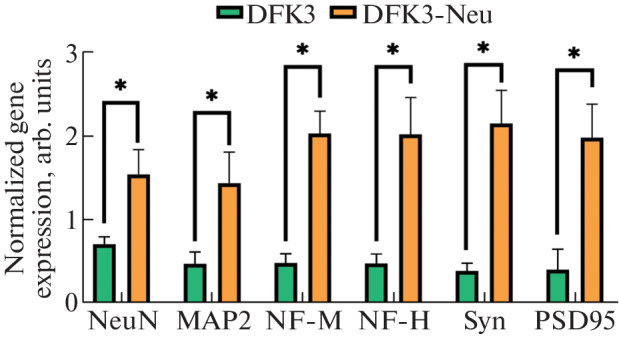
Analysis of neuronal markers in DFK3 and DFK3-Neu cells. Differences from control are significant (*) at *p* < 0.05 (Mann–Whitney test).

At the next stage, we studied the effect of the CSF of injured animals on the survival of neuronal cells. For this purpose, we made TBI in rats according to the previously described protocol [8]; 9 days after TBI, CSF was taken from the anesthetized animals through the foramen magnum. Then, we cultivated DFK3-Neu cells in the presence of CSF of injured (CSF-TBI) or healthy (CSF-norm) animals. The ratio of CSF and differentiation medium was 1 : 1. After 12, 24, or 48 h of cultivation, the viability of neurons was assessed using the MTT asssay (assessment of dehydrogenase activity according to Mossman) [12]. We found that culturing neurons with CSF from injured animals resulted in a decrease in cell viability compared to culturing in the presence of the control CSF (Fig. 2). After 12 h of cultivation, the difference was 11%; after 24 h, 14.6%; and after 48 h, 20.9%.

**Fig. 2.**
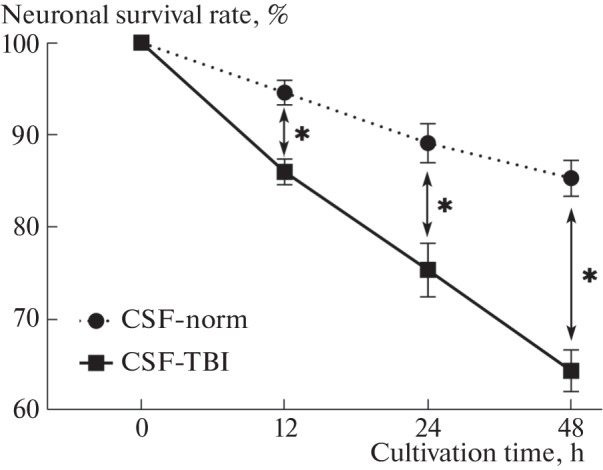
Comparison of the viability of DFK3-Neu cells cultured in the presence of CSF of injured (CSF-TBI) and non-injured (CSF-norm) rats. Differences from control are significant (*) at *p* < 0.05 (Mann–Whitney test).

As part of the development of this model, it was necessary to test it to make sure that DFK-Neu cells are able to respond to drug treatment. For this purpose, we used pyrrolylazine and indolylazine derivatives synthesized by us [13, 14], which can induce accumulation of chaperones in cells and were shown to have a neuroprotective effect in models of secondary damage after TBI [8] and in Alzheimer’s disease [14]. DFK3-Neu cells were cultured for 24 h in the presence of PQ-29 (a pyrrolylazine derivative) and IA-50 (an indolylazine derivative) at concentrations of 0.5, 2, and 8 μM. One day later, the cells were lysed, and the lysates were subjected to Western blot analysis according to the previously described protocol [14]. The blot was sequentially incubated with mouse antibodies against Hsp70 clone 3C5 [11], GAPDH (clone 6C5, Abcam, United Kingdom) and then with horseradish peroxidase-conjugated anti-mouse antibodies (Abcam, United Kingdom). Blot staining with the antibodies against GAPDH was used as a control for the total protein load of the samples. The result of blot hybridization with antibodies is shown in Fig. 3a. On the basis of three independent experiments using the TotaLab Quant software, the intensity of the protein zones was digitized. The result of digitization is shown in Fig. 3b as the normalized ratio of the intensity of Hsp70 zones to the intensity of the reference protein GAPDH. We showed that PQ-29 and IA-50 at a concentration of 8 μM caused a 2.3- and 2.2-fold increase, respectively, in the amount of Hsp70 protein in rat neurons. Fig. 4

**Fig. 3.**
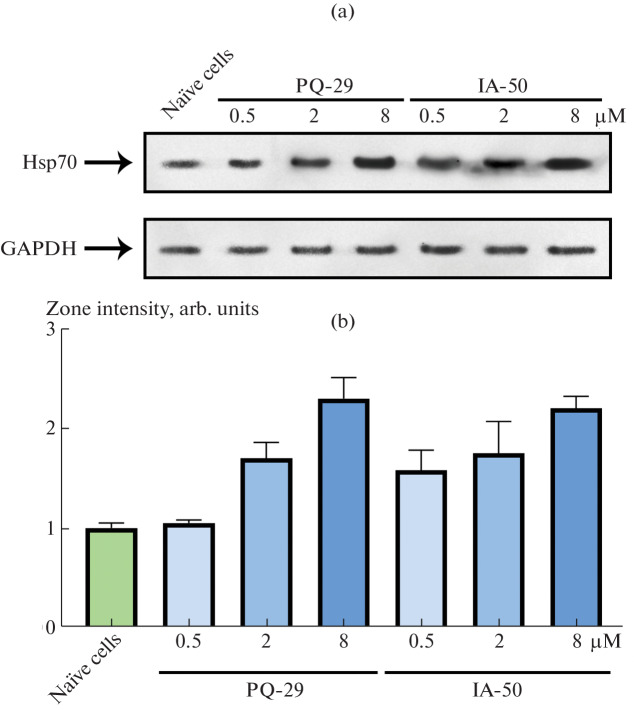
Effect of compounds PQ-29 and IA-50 on the content of Hsp70 protein in DFK3-Neu cells. (a) Representative results of Western blot analysis are shown. Anti-GAPDH antibodies were used as load control. (b) The result of digitization of the intensity of the blot zones, presented as the ratio of the intensity of the Hsp70 zones to the intensity of the reference protein GAPDH normalized to the value obtained for the untreated cells.

**Fig. 4.**
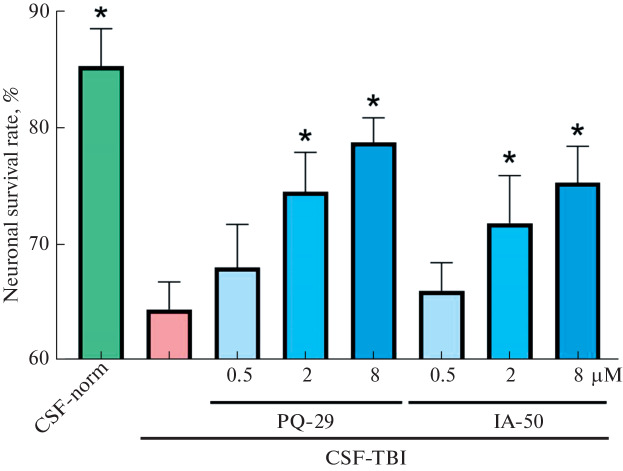
The use of compounds PQ-29 and IA-50 prevents the death of DFK3-Neu cells caused by cultivation in the presence of CSF of injured rats. CSF-norm, cells cultured for 48 h in the presence of CSF of healthy animals; CSF-TBI, cells cultured for 48 h in the presence of CSF of injured rats. Differences from control (CSF-TBI) are significant (*) at *p* < 0.05 (Mann–Whitney test).

The key stage of this study was the verification of the new model. To do this, we assessed the survival of rat neurons in the presence of CSF of injured animals, as well as neuroprotective compounds from the classes of pyrrolylazines and indolylazines using the MTT assay. We found that PQ-29 and IA-50 effectively prevented neuronal death in the presence of CSF of rats after TBI. At the highest tested concentrations (8 μM), these compounds prevents the death of 14.36% and 10.9% of the cell population (for PQ-29 and IA-50, respectively).

Thus, we have developed a new convenient model for assessing secondary injuries after TBI. The key feature of the presented model is that it makes it possible to evaluate the cytotoxic effect of the CSF of injured rats on cultured rat cells with a neuronal phenotype. To verify the relevance of the new model, we tested the cytoprotective activity of compounds from the classes of pyrrolylazines and indolylazines, which we previously reported as neuroprotectors. Both compounds demonstrated protective activity in our in vitro secondary injury model. Compound IA-50 was tested in the model of secondary injuries after TBI for the first time, and its therapeutic effect was detected. A certain limitation of the possible use of this model is the lack of data on the possibility of using it to test drugs that do not affect the chaperone system.
